# Supplementary material for: A Critical, Nonlinear Threshold Dictates Bacterial Invasion and Initial Kinetics During Influenza
Source: Sci Rep. 2016 Dec 15;6:38703. doi: 10.1038/srep38703 (PMC5156930; doi:10.1038/srep38703)
Supplement: Supplementary File [file srep38703-s1.pdf]

Supplementary Information

A Critical, Nonlinear Threshold Dictates Bacterial Invasion and Initial Kinetics During Influenza

Amber M. Smith[1,*], Amanda P. Smith[1]

[1]Department of Infectious Diseases, St. Jude Children's Research Hospital, Memphis, TN 38105, USA

*Email: amber.smith@stjude.org

Influenza-Pneumococcal Coinfection Model Schematic, Fits, and Parameters

The coinfection model schematic is shown in Figure S1. Model fits to lung viral and bacterial titers from groups of mice infected 7d after influenza A/Puerto Rico/8/34 (H1N1) (PR8) with PBS (black line) or pneumococcal strain D39 (red line) are shown in Figure S2 and the model parameters are in Table S1 (Smith et al., 2013).

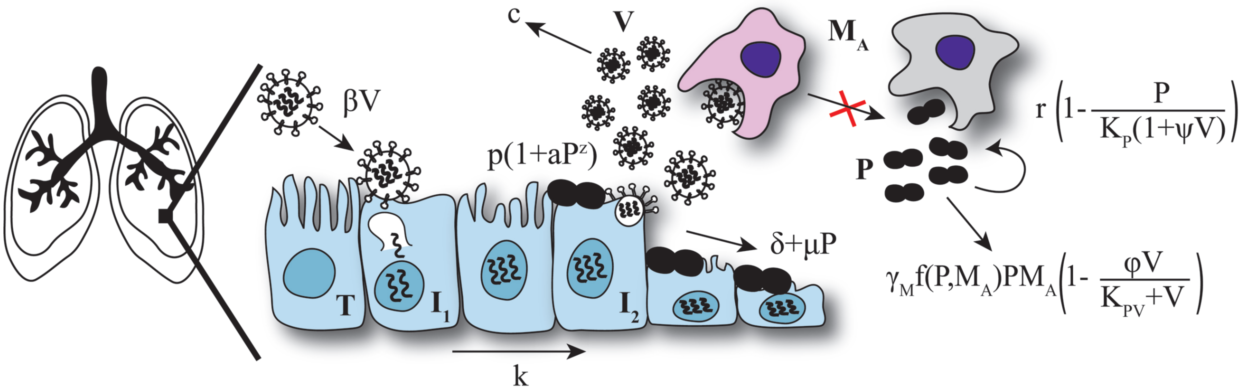

Figure S1: Schematic of the coinfection model (Smith et al., 2013). Target cells (T) are infected with virus (V) at rate βV. Infected cells enter an eclipse phase (I_1) and transition to producing virus (I_2) at rate k. Productive infected cells (I_2) produce virus at rate p and removed at rate δ. Bacteria (P) replicate logistically with maximal rate r and carrying capacity K_P, which is increased by ψV when virus is present. Bacteria are phagocytosed by alveolar macrophages (M_A) at rate $\gamma_M f(P, M_A)$, which is decreased by $\phi V/(K_{PV} + V)$ when virus is present. In the presence of bacteria, infected cells are killed at rate μP and virus production/release is increased by aP^z.

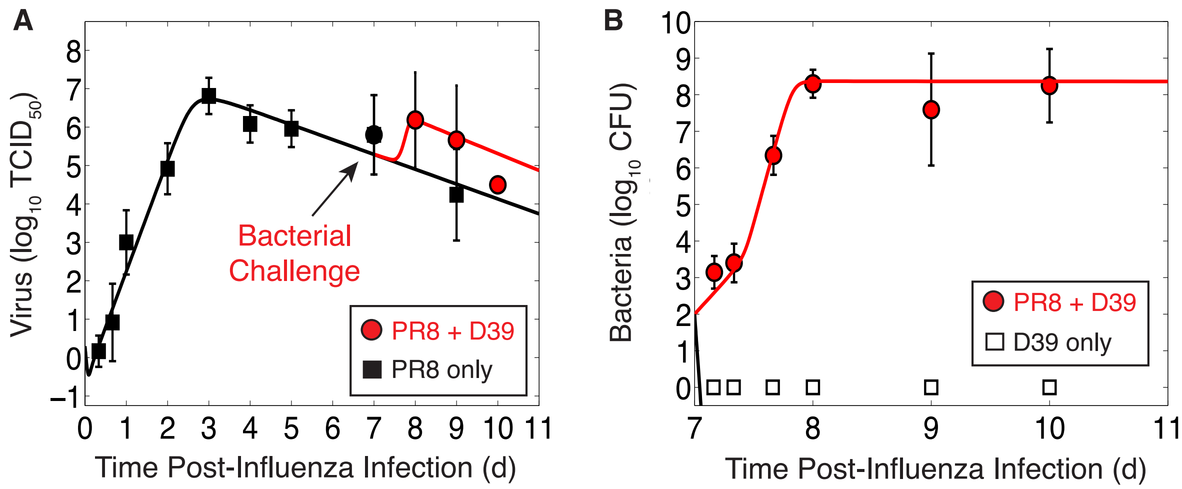

Figure S2: Coinfection model fit to lung titers of mice coinfected with PR8 and 10^3 **CFU D39 (Smith et al., 2013).** Fit of the coinfection model (Equations (2)-(6) (main text)) to viral (panel A, red) and bacterial (panel B, red) lung titers from individual mice infected with 10^2 $TCID_{50}$ PR8 virus followed 7 days later by 10^3 CFU pneumococcal strain D39. Solid black lines are the model solutions with $P = 0$ (panel A) or $V = 0$ (panel B). Parameters for the model curves are in Table S1.

Datasets With Heterogeneous Bacterial Titers

Our results suggest that heterogenous bacterial titers indicate an AM:dose ratio that lies close to or below the initial dose threshold. This heterogeneity lessens with increased dose or AM depletion. Two datasets (Figure S3) in our previous work exhibited heterogenous behavior (Smith et al., 2013). In the first dataset (Figure S3A), mice were infected with 10^2 $TCID_{50}$ PR8 followed by 10^2 CFU D39 7d pii. At both 24h and 48h pbi, bacterial titers split into two groups where ~50% at high levels and ~50% at low levels. Comparing these data with data from mice infected with 1 $\log_{10}$ higher bacteria (10^3 CFU, Figure S2B) shows that decreasing the dose increases the heterogeneity. These data suggest that the lower dose (10^2 CFU) is close to the initial dose threshold and that the higher dose (10^3 CFU) is sufficiently above the threshold.

In the second dataset (Figure S3B), mice were infected with 10^2 $TCID_{50}$ PR8-PB1-F2 (1918) followed by 10^3 CFU D39 7d pii. At 8h and 16h pbi, some mice begin to clear the inoculum while others have bacterial titers that are 1-2 $\log_{10}$ higher than the inoculum. At 16h pbi, two distinct groups emerge such that 50% have low bacterial loads and 50% have high bacterial loads. Comparing these data with data from mice infected with the PR8 virus and the same bacterial dose (10^3 CFU, Figure S2B) shows that altering the viral strain can change the heterogeneity and trajectory of bacterial titers. Our results here suggest that infection with the PR8-PB1-F2(1918) virus yields an AM:dose ratio closer to the threshold than the PR8 virus. Thus, we hypothesize that infection with PR8-PB1-F2(1918) results in less AM depletion. Although the extent of AM depletion is unknown for the PR8-PB1-F2(1918) virus, a lower viral titer 7d pii compared to infection with PR8 was observed (Figure S3B inset) (Smith et al., 2011a, 2013) and may indicate reduced AM depletion.

Table S1: Parameter values of the influenza model (Smith et al., 2011a), pneumococcal model (Smith et al., 2011b), and coinfection model (Equations (2)-(6) (main text)) (Smith et al., 2013).

	Parameter	Description	Value	Units
**Influenza A Virus**	β	Virus infectivity	2.8×10^{-6}	$(\text{TCID}_{50})^{-1}\text{day}^{-1}$
	k	Eclipse phase	4.0	day^{-1}
	δ	Infected cell death	0.89	day^{-1}
	p	Virus production	25.1	$(\text{TCID}_{50})\text{day}^{-1}$
	c	Virus clearance	28.4	day^{-1}
	$T(0)$	Initial uninfected cells	10^7	cells
	$I_1(0)$	Initial infected cells	0	cells
	$I_2(0)$	Initial infected cells	0	cells
	$V(0)$	Initial virus	2.0	TCID_{50}
**Pneumococcus**	r	Bacterial growth rate	27.0	day^{-1}
	K_P	Carrying capacity	2.3×10^8	CFU
	γ_{M_A}	Phagocytosis rate	1.35×10^{-4}	$\text{cell}^{-1}\text{day}^{-1}$
	n	Maximum bacteria per AM	5.0	$(\text{CFU})\text{cell}^{-1}$
	M_A^*	Number of AMs	10^6	cells
	$P_0(7)$	Initial bacteria at day 7 post-influenza	10^3	CFU
**Coinfection**	ϕ	Decrease in phagocytosis rate	0.87	
	K_{PV}	Half-saturation constant	1.8×10^3	TCID_{50}
	a	Increase in virion production/release	1.2×10^{-3}	$(\text{CFU})^{-z}$
	z	Nonlinearity of virion production/release	0.50	
	ψ	Increase in carrying capacity	1.2×10^{-8}	$(\text{TCID}_{50})^{-1}$
	μ	Toxic death of infected cells	5.2×10^{-10}	$(\text{CFU})^{-1}$

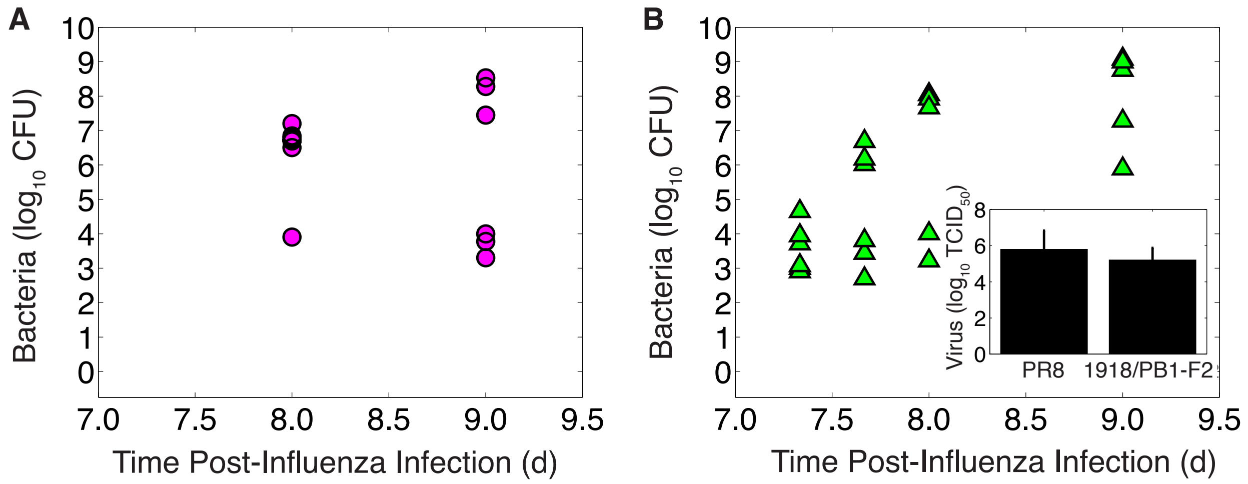

Figure S3: Dichotomous Bacterial Titers (Smith et al., 2013). (A) Bacterial titers for infection with 10^2 $TCID_{50}$ PR8 followed by 10^2 CFU D39 7d pii. (B) Bacterial titers for infection with 10^2 $TCID_{50}$ PR8-PB1-F2(1918) followed by 10^3 CFU D39 7d pii. Inset: Viral titers 7d pii for infection with 10^2 $TCID_{50}$ PR8 or PR8-PB1-F2(1918) (Smith et al., 2011a, 2013).

Gating Strategy for Flow Cytometric Analysis

Figure S4 shows the gating strategy used to define AMs in Figure 5 in the main text. Data shown are from a naive mouse.

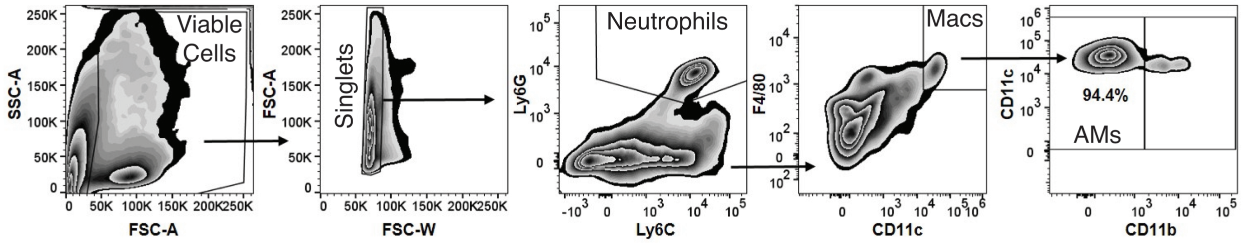

Figure S4: Gating Strategy to Define AMs. Viable cells are gated from a forward scatter/side scatter plot then singlet inclusion. Neutrophils (Ly6G^{hi}) are then gated out and macrophages (CD11c^{hi}F4/80^{hi}) are gated then AMs subgated as CD11b^{-}.

References

Smith, A.M., Adler, F.R., McAuley, J.L., Gutenkunst, R.N., Ribeiro, R.M., McCullers, J.A., and Perelson, A.S. Effect of 1918 PB1-F2 expression on influenza A virus infection kinetics. *PLoS Comput. Biol.*, 7(2):e1001081, 2011a.

Smith, A.M., McCullers, J.A., and Adler, F.R. Mathematical model of a three-stage innate immune response to a pneumococcal lung infection. *J. Theor. Biol.*, 276(1):106 – 116, 2011b. doi: 10.1016/j.jtbi.2011.01.052.

Smith, A.M., Adler, F.R., Ribeiro, R.M., Gutenkunst, R.N., McAuley, J.L., McCullers, J.A., and Perelson, A.S. Kinetics of coinfection with influenza A virus and *Streptococcus pneumoniae. PLoS Pathog.*, 9(3):e1003238–e1003238, 2013.
